# Preprocessing 2D data for fast convex hull computations

**DOI:** 10.1371/journal.pone.0212189

**Published:** 2019-02-22

**Authors:** Oswaldo Cadenas, Graham M. Megson

**Affiliations:** 1 School of Engineering, London South Bank University, London, United Kingdom; 2 School of Electronics and Computer Science, University of Westminster, London, United Kingdom; University of Michigan, UNITED STATES

## Abstract

This paper presents a method to reduce a set of *n* 2D points to a smaller set of *s* 2D points with the property that the convex hull on the smaller set is the same as the convex hull of the original bigger set. The paper shows, experimentally, that such reduction accelerates computations; the time it takes to reduce from *n* down to *s* points plus the time of computing the convex hull on the *s* points is less than the time to compute the convex hull on the original set of *n* points. The method accepts 2D points expressed as real numbers and thus extends our previous method that required points as integers. The method achieves a percentage of reduction of points of over 90% in a collections of four datasets. This amount of reduction provides speedup factors of at least two for various common convex hull algorithms. Theoretically, the reduction method executes in time within *O*(*n*) and thus is suitable for preprocessing 2D data before computing the convex hull by any known algorithm.

## Introduction

The convex hull of a set of points in two dimensions (2D) gives a polygonal shape as a visual indication of the smallest region containing all the points. It is used as the base for other geometric algorithms; it is always found as a function in computational geometry software packages [1]. Reports of applications of the convex hull continue to appear in different areas such as zoology [2] and immunoinformatics [3].

Discarding points that cannot be part of a 2D convex hull, before computing it, has proven to be popular and useful as a preprocessing step. Indeed, CGAL [4], a popular package for performing computational geometry tasks, includes the convex hull function ch_akl_toussaint that has embedded into it a simple method for discarding 2D points, dating from 1978 [5]. This preconditioning method is linear in time but only discards around half the points. In previous work [6], it was observed that the greater the reduction of points the greater the speedup that is achieved when computing the convex hull; only that method requires integer points. The new method presented here accepts points expressed as real numbers. The method in [5], uses four extremal points to create a region from where points are discarded. The preprocessing method here, drawing on from our previous work [6], aggressively seeks to discard more points by quickly identifying many more regions where to operate. On each region, a one-step test decides which points to keep or remove. As a result, the percentage of reduction of points seen in four datasets is of over 90%, reaching up to 98% at instances. With this amount of reduction, speedups of at least a factor of two were measured when computing the convex hull via this proposed preprocessing method (compared to the computation without the preprocessing using the same algorithm for the convex hull). The main features of the new preprocessing method are:

- We prove the method is linear, with a computational time within *O*(*n*). This is key to provide computation speedups for state-of-the-art convex hull algorithms.
- No explicit sorting of points is required at any step of the preprocessing.
- The method discovers parallel work in its internal processing. The results reported in this paper do not exploit this parallelism, however. This new found parallelism can be exploited leading to even further accelerations (to be reported elsewhere).

The first two bullet points are key for the method to be useful. The best known general convex hull algorithm is of time complexity *O*(*n* lg *h*) (lg *n* denotes log_2_ *n* throughout) where *h* is the number of points in the convex hull (*h* is unknown from the outset) [7]; any preconditioning method shall be of at most that same complexity. The proposed method remains simple and practical. The preprocessing takes a pointer to the points and its size *n* and returns the cardinality of the reduced subset *s* and an array containing the indices of points of the reduced subset. This simplicity plus its time complexity of *O*(*n*) makes the method suitable for being integrated into any existing computational geometry package.

The paper starts by explaining the processing steps of the method and proving that the method is linear. It follows giving a heuristic to determine a parameter *m*, the number of regions on where to operate, that leads to speedups greater than 1. Determining to which region a point belongs to in constant time is fully developed. This was a challenge since this is a statistical binning problem with a typical cost of *O*(*n* lg *m*) time for binning data when bins are of a different sizes. The paper gives a solution to the data binning, as it emerges in the proposed method for this problem, in *O*(*n*) time. This is an extra contribution of this work. The experiments carried out here use the same four datasets used in our previous work [6]. Similarly, convex hull algorithms available from standard packages such as CGAL and SciPy [8] (both of best case time complexity of *O*(*n* lg *n*)) were used when benchmarking speedup benefits of the preprocessing in the computation. In spite of the algorithm of Chan [7] being the best known convex hull algorithm, it is not found in common computational geometry packages and so the paper includes results using an ad-hoc implementation of Chan’s algorithm.

## Materials and methods

### Proposed preconditioning method

We assume a set of 2D points with (*x*, *y*) coordinates; *x*, *y* as real numbers. This set is the input to the convex hull problem. The cardinality of this set, *n*, is not known a priori. For the purpose of computation, all input points reside in an array *P* = [*X*_*C*_, *Y*_*C*_] with *X*_*C*_ and *Y*_*C*_ as vectors of length *n* corresponding to the *x*-coordinate and *y*-coordinate of all points in *P* respectively. Values in vectors *X*_*C*_, *Y*_*C*_ may appear in arbitrary order and so they are not sorted. Each *x* and *y* coordinate value is expressed in either single or double precision floating-point format.

**Fig 1:**
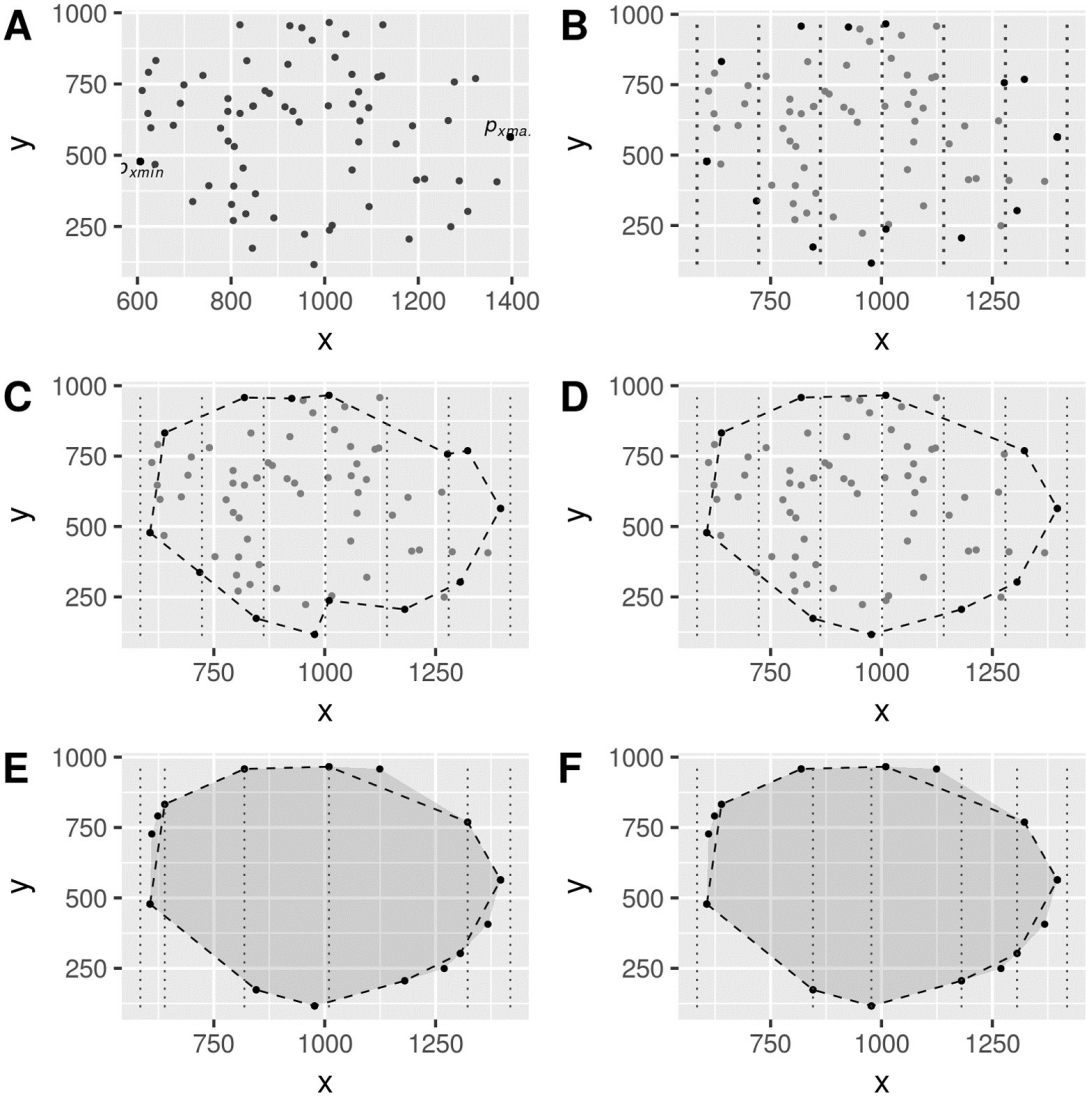
Steps of the proposed preprocessing method for reducing points. A: Step 1 finds *p*_*xmin*_ and *p*_*xmax*_ values and points. B: Steps 2 and 3 of quantization of points into bins and points with minimum and maximum *y*-coordinate per bin. C: Step 4 creates the upper and lower fence. D: Step 5: get convex lower and upper fences. E: Step 6 finds points outside the upper and lower fences. F: An example of bin boundaries moved according to lower fence points.

A convex hull algorithm finds a polygon *CH* of *h* points derived from processing all points from the input set *P*. The proposed method takes as input the set of *n* original points in *P* and outputs a subset of points *P*_*S*_, of *s* points, such that the convex hull of *P*_*S*_ is also *CH*. The proposed preprocessing method is given as a number of contiguous steps. These are as follows:

1. Find *n*, *p*_*xmin*_ = *min*(*X*_*C*_), *p*_*xmax*_ = *max*(*X*_*C*_) from *P*. This is illustrated in Fig 1A. Note *min*(*X*_*C*_), *max*(*X*_*C*_) are values, not points. If there are multiple points having their *x*-coordinate equal to these extreme values, we keep any one of those points. These two extreme points *p*_*xmin*_ and *p*_*xmax*_ correspond to the points with smallest and greatest *x*-coordinate values of the set *P*.
2. Given a constant parameter *m*, quantize the points of *P* according to the *x*-coordinate value. This quantization divides the range of values of vector *X*_*C*_, that is the range *max*(*X*_*C*_) − *min*(*X*_*c*_), into *m* equally-sized intervals. This is a form of binning each point into *m* bins, so that, each point is tagged with integer values 1, …, *m* corresponding to the bin where its *x*-coordinate value lies. The choice of parameter *m* is discussed later.
3. Find the two points in each bin whose *y*-coordinate values are maximum and minimum. Step 2 and step 3 are easily understood graphically as shown in Fig 1B. With these two points per bin, we form two vectors of points, each of size *m*.
4. Starting with the point at *p*_*xmin*_, join the points across all the *m* bins whose *y*-coordinate is minimum, ending with point at *p*_*xmax*_. These line segments form a lower envelope of size *m* + 2 points and it looks like a fence, or lower fence. Similarly, we build an upper fence also starting with point at *p*_*xmin*_ and ending with point at *p*_*xmax*_ connecting the points whose *y*-coordinate is a maximum across all bins. These line segments also connect *m* + 2 points. Notice these two fences are polylines; the points are sorted according to its *x*-coordinate value due to the nature of the bin intervals. The upper and lower polylines fences are shown in Fig 1C.
5. Remove non-convex points from the lower and upper fences. Note that in Fig 1C both the lower and the upper fences have points which make the polylines non-convex; we remove those points to make the fences convex polylines. Note that the method in this step deals with a small subset of points, of size *m* + 2, for each one of these two fences. Fig 1D shows the convex fences after this quick removal step. All points from each convex fence polyline are included in the output set *P*_*S*_.
6. Find all points in *P* that are outside the convex upper and convex lower fences and include them in the output set *P*_*S*_. These points are shown in Fig 1E along with the convex hull computed from the original points *P*. Note that, in essence, all points inside the region formed by the lower and upper fence have been removed from input *P*. Also, note that the boundaries of the bins in Fig 1E have moved to coincide with the points of the upper fence. This is most convenient when finding points outside the upper fence. Similarly, for finding the points outside the lower fence, the bin boundaries are moved as shown in Fig 1F. This rebinning of points is key for the method to be of linear time, and thus is addressed in detail later in a separate section.

### The proposed method runs in *O*(*n*) time

Step 1 of the method requires seeing all points of *P* once and it is of *O*(*n*) time. In Step 2, each point in *P* is quantized in constant time, according to its *x*-coordinate value, since we assume the number of intervals to be *m* (known value) and all intervals are of equal size. As a small example, suppose the *x*-coordinate values of *p*_*xmin*_ and *p*_*xmax*_ as 2.3 and 9.1 respectively with *m* = 4. The boundaries of the intervals are {2.3, 4.0, 5.7, 7.4, 9.1}, that is an interval size of 1.7 (calculated as (9.1-2.3)/4). Determining to which interval a given (*x*-coordinate) value belongs to inside these interval boundaries is a trivial calculation; thus Step 2 takes *O*(*n*) time.

Step 3 uses the quantized value for each point from Step 2 to find points with extreme *y*-coordinate values on each bin. Say we keep array *L*[*j*] for *j* = 1, …, *m* to record points with the minimum *y*-coordinate for each bin. When seeing point *p*_*i*_ = (*x*_*i*_, *y*_*i*_) for any *i* = 1, …, *n*, if in Step 2 the quantized value of *p*_*i*_ is *b* then the array is updated as *L*[*b*] = *min*(*L*[*b*], *y*_*i*_). Thus, this Step 3 is done in *O*(*n*) time for the set *P* by visiting each point once. Note that finding the maximum *y*-coordinate value in each bin is also performed in the same pass along *P*.

Step 4 does the work of creating a polyline; this is straightforward and of time *O*(*m*). Step 5 makes the lower and upper fence to be of convex line segments. As these are polylines of size *m* + 2, we could ultimately use Melkman [9] to complete this task in time *O*(*m*). Working on the lower and upper fence can be done concurrently and so there is some level of parallelism made explicit in this step. Parameter *m* is either given as a constant or we will make *m* ≪ *n*. All that remains to be shown is that Step 6 can run in *O*(*n*) time. We discuss Step 6 separately below.

#### Step 6: Keeping points outside lower and upper fences

**Fig 2:**
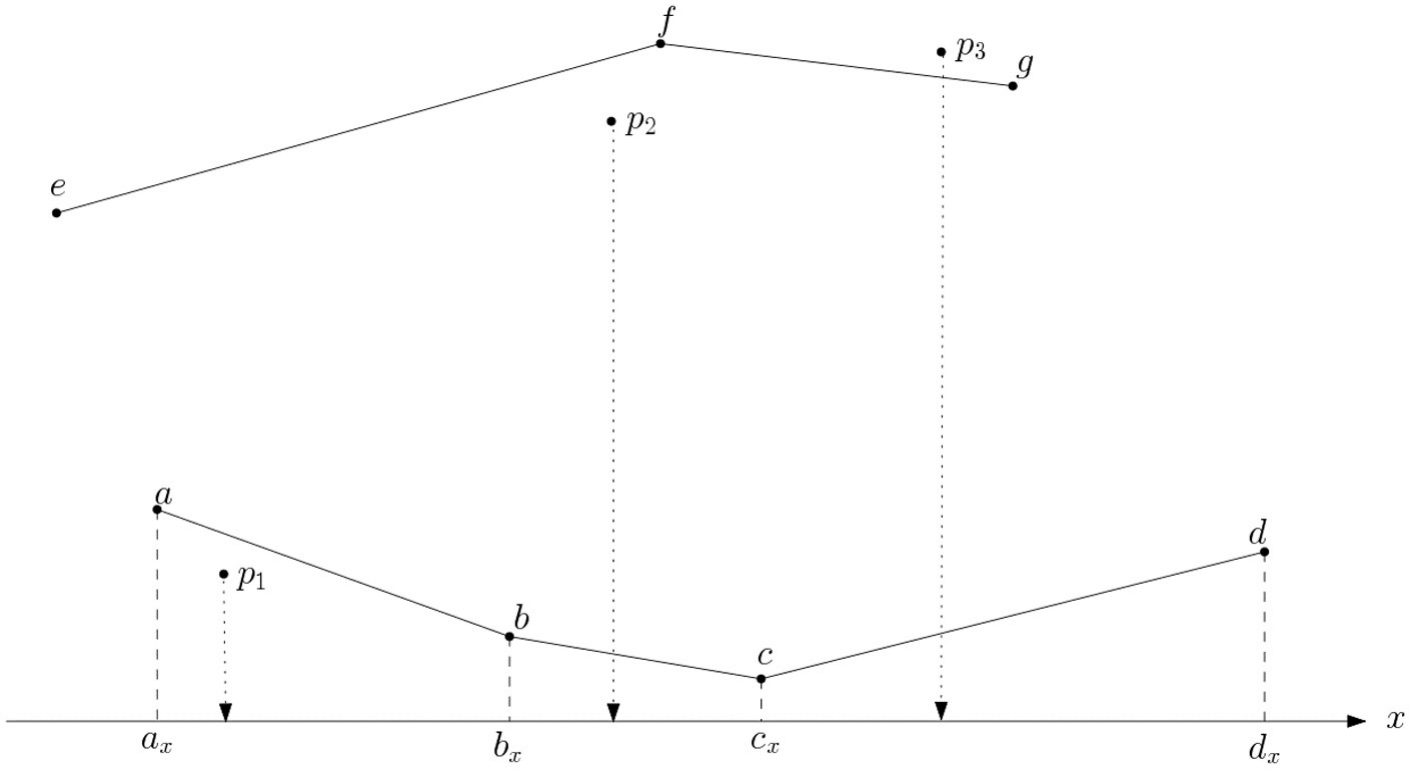
Three points to be managed by Step 6 of the preprocessing. Lower fence is composed of points *a*, *b*, *c*, *d* and upper fence by points *e*, *f*, *g*. For each point *p*_1_, *p*_2_, *p*_3_ we need to find whether or not they are outside either of the two fences. When checking points against the lower fence, interval boundaries along the *x* axis are aligned with the lower fence.

The operation performed by Step 6 is simple. Consider the situation for three points *p*_1_, *p*_2_, *p*_3_ and the lower and upper fence as shown in Fig 2. We run two checks: one for checking whether points are outside the lower fence and one for checking whether they are outside the upper fence (these could be executed in parallel).

For example, for checking whether points are outside the lower fence, we set up new interval boundaries by projecting the *x*-coordinate of the lower fence points *a*, *b*, *c*, *d* into the *x* axis. Then, for point *p*_1_, we determine that it belongs to the bin spanning from boundary values from *a*_*x*_ to *b*_*x*_. A simple test, based on the cross-product of *a*, *b*, *p*_1_ will determine whether *p*_1_ is outside the lower fence in Fig 2. This is equivalent to finding if point *p*_1_ is on the right or left of the line segment $\bar{ab}$. That is, we set new bins along the *x* axis, and all points of *P* whose *x*-coordinate value lies on the same bin are checked against a single line segment; we check whether these points are on the right or left of this unique line. The computation keeps (in *P*_*s*_) those points that are on the right of the line segments used in the cross-product check. Note that this step has made explicit a great deal of parallelism. The number of line segments of the upper and lower fences combined, is as many independent tasks that can be executed in parallel.

When deciding whether to keep point *p*_3_, for example, we check to see whether it is on the right of the line segment $\bar{gf}$ in Fig 2; in such a case it is outside the upper fence.

**Fig 3:**
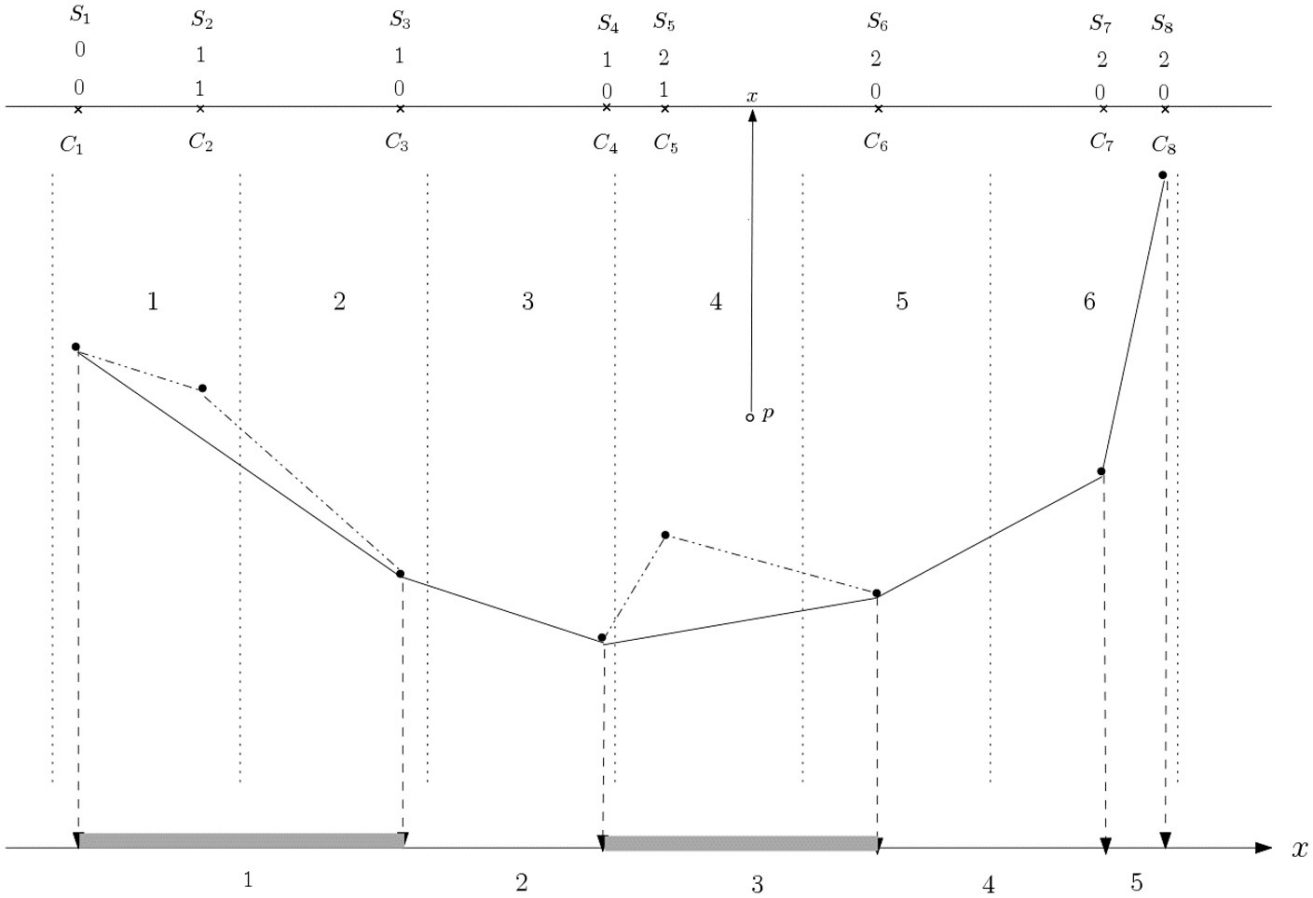
New bins intervals derived from a lower fence. For an original *six* bins of equal size intervals (dotted vertical lines), the polyline joining the points with minimum *y*-coordinate values on each bin was processed into a convex polyline. The points of the convex polyline are projected into the *x* axis to form *five* new bins; these are of non-equal sizes.

Although all above seems simple at first, determining the new bin for a point in Fig 2 is the challenge in this step. The challenge is to determine it fast. Note that all points were previously quantized (Step 2) into *m* bins that were all of equal size intervals. We observe two things that change when the bin boundaries are moved according to the projection of the lower/upper fence points as shown in Fig 2. First, the bins are now of different interval sizes and secondly, the number of bins might be less than *m*. These two observations are clearly shown in Fig 3, using a lower fence as an instance.

The idea pursued in this work is to reuse the quantized values for points as computed back in Step 2 to determine the new quantized values in this Step 6. We will see that each point is re-binned into the new bins in constant time. From the same Fig 3 we see that the points located in the new Bin 1 interval (shaded at the bottom) could have originally been quantized as either 1 or 2. Similarly, points located within the new Bin 3 interval (also shaded at the bottom) could have originally been quantized as either 3, 4 or 5. There is an easy way to determine the new quantized value for a point (according to the new bin intervals in Step 6) by using the information of the previous quantized value for the same point computed back in Step 2.

The idea is shown at the top of Fig 3. First we project the points of the polyline found in Step 4 onto the *x* axis (marked with the cross symbol ×), say into vector *C*. Then we annotate a 1 on each position where the point has been removed from this polyline in Step 5 (at *C*_2_, *C*_5_) and perform a prefix sum over *C* into vector *S*. Observe the prefix sum takes *O*(*m*) time. Consider the point *p* in Fig 3 with a projected value of *x* into the *x*-axis as shown. Point *p* was originally quantized as belonging to bin 4. Note that as *x* > *C*_5_, then point *p*, now quantized according to the new bins, is of value 5 − *S*_5_ = 5 − 2 = 3. Note, that should the *x*-coordinate of point *p* been located between *C*_4_ and *C*_5_ the new bin value would have been of 4 − *S*_4_ = 4 − 1 = 3; this is also clear from the figure. In general, this re-binning of points is presented as Algorithm 1.

**Algorithm 1**: Rebinning of points

**input**: An array *P* of *n* points as (*x*, *y*) (reals); an array *Q* of original quantized values for *P* (integers); an array *C* of size *m* + 2 with boundary interval values for the bins based on fences (reals); an array *S* of size *m* + 2 with a prefix sum of removed non-convex points from fences (integers)

**output**: Array *Q* updated with quantized values for the new bins based on convex fences

**for** *i* = 0 **to** *n* **do**

 *x*, *y* = *P*[*i*];

 *q* = *Q*[*i*];

 **if** *x* ≥ *C*[*q* + 1] **then**

  *Q*[*i*] = *Q*[*i*] + 1 − *S*[*q* + 1];

 **else**

  *Q*[*i*] = *Q*[*i*] − *S*[*q*];

 **end**

**end**

From the pseudo-code it is clearly seen that rebinning the quantized values for each point takes *O*(1) time and thus an overall *O*(*n*) time to quantize all points according to the new bins of different interval sizes. Binning data for a number of bins of different interval sizes, typically takes *O*(lg *m*) time per value using binary search [1]; this results in a global *O*(*n* lg *m*) time for *n* values. This cost is avoided here by reusing the previous quantization values for the points. More on this key issue is postponed to the discussion section.

Once the points have been rebinned, keeping or rejecting points is straightforward. The procedure is shown in Algorithm 2. The procedure is applied as a function to each fence independently (input *C* is different for each fence) and so the output set *P*_*s*_ is the union of the output arrays *PR* while *s* is the sum of the returns *nr*. The function *IsLeft*(*p*1, *p*2, *p*) is a cross product test. Thus deciding whether a point shall be accepted or rejected is performed in constant time resulting in a global computational time *O*(*n*) for the whole set *P* in Step 6.

**Algorithm 2**: Procedure to keep points for a given convex fence

**input**: An array *P* of *n* points as (*x*, *y*) (reals); an array *Q* of new rebinned values for *P* (integers); an array *F* of size *O*(*m*) with the convex points of a fence (reals)

**output**: Array *PR* with points to be kept outside the fence of size *nr*

*nr* = 0;

**for** *i* = 0 **to** *n* **do**

 *p* = *P*[*i*];

 *q* = *Q*[*i*];

 *p*1 = *F*[*q*];

 *p*2 = *F*[*q* + 1];

 **if** *not IsLeft*(*p*1, *p*2, *p*) // *p* is to the right of line *p*1 → *p*2

 **then**

  *PR*[*nr*] = *p*; // Keep point *p*

  *nr* = *nr* + 1;

 **else**

  // Point *p* is rejected

 **end**

 **return** *nr*;

**end**

All steps, from Step 1 to Step 6 have an upper bound in computational time of *O*(*n*) and thus the preconditioning method proposed here can be applied to any known convex hull algorithm without any detriment to the computational complexity of the algorithm.

### Choosing parameter *m*

We just stated that the value of *m* is a constant; and probably a small value compared to the input size *n*. Without assuming any statistical distribution for the input set of points *P*, it is not easy to determine an optimal value for this parameter. For the method to be practical, we would like to choose a value of *m* that provides the greatest acceleration when computing the convex hull of the input *P* using known algorithms. It would not be sensible to seek only for a value of *m* that maximimizes the greatest reduction of points. Thus, this paper takes an empirical approach to suggest a suitable value of *m*.

**Fig 4:**
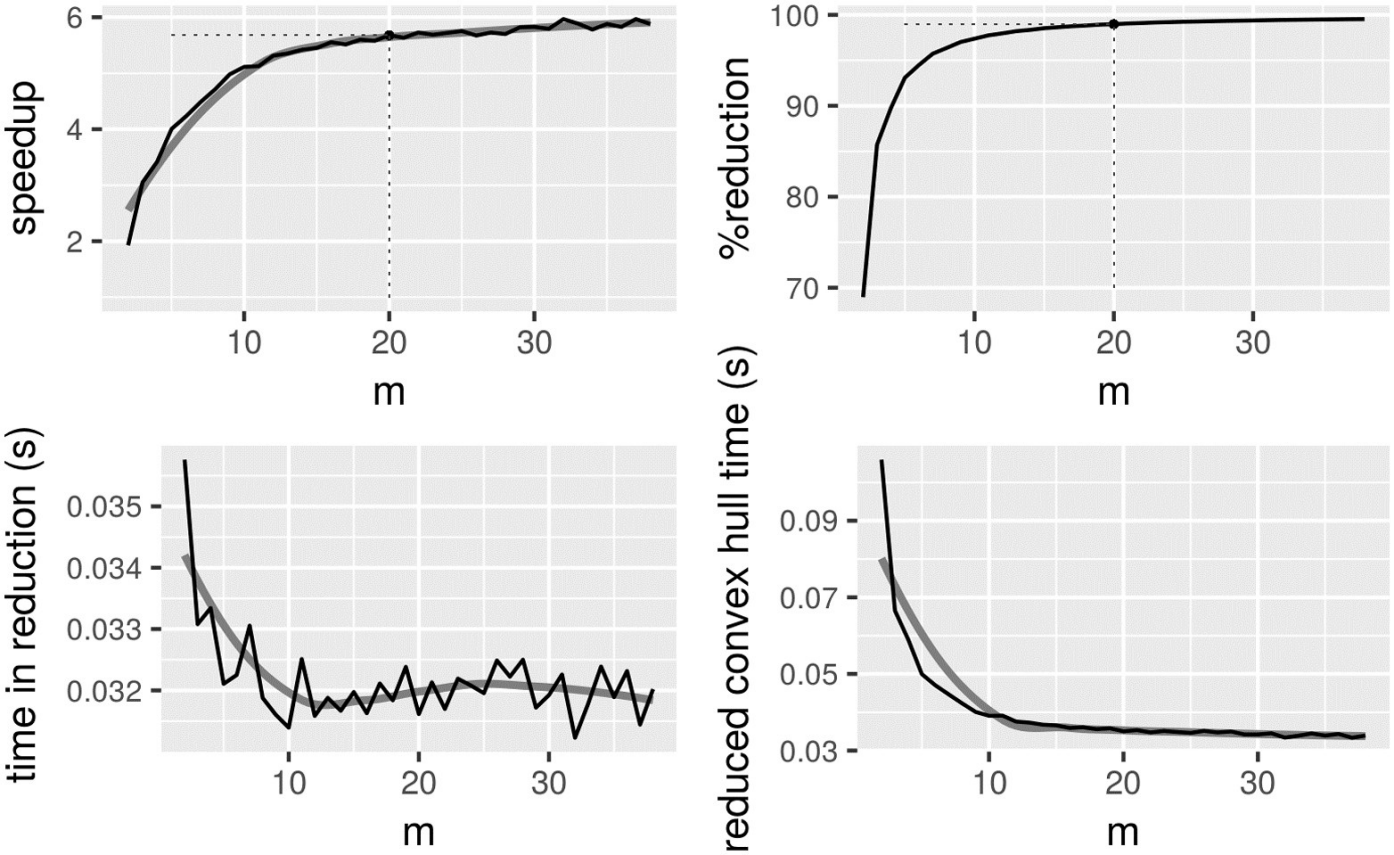
Preprocessing method as a factor of parameter *m* with *n* constant. Upper left: Speedup gained by reducing the *n* points first to *s* points and then computing the convex hull on *s* points. Upper right: Percentage of reduction of points. Lower left: Time spent reducing the points in seconds. Lower right: Time reducing the points plus convex hull time on the reduced set in seconds. The grey lines are a visual aid to the reader of the underlying pattern shown by the plot. The values for ⌈*m* = lg *n*⌉ are marked.

Fig 4 was generated keeping *n* size constant while parameter *m* changes from the value of 2 to the value ⌊2 lg *n*⌋. We kept *n* to 1 million points and a number of runs were performed with random points, uniformely distributed, inside a convex superellipse: we controlled the superellipse parameters to keep the shape from a rhombus shape to a rectangle shape with rounded corners. We have chosen a uniform distribution as it provides a well behaved scenario in which all vertical regions delimited by the bins will have points in the lower and upper fences. In general, this is a difficult problem that depends on the distribution of points. From this simple experiment, we derive a simple rule for choosing *m*. This rule is applied, in the results section, to real datasets. The results show that the rule is not only practical but useful.

The convex hull algorithm used in Fig 4 is the ch_bykat from the CGAL package [4]; this has an expected time complexity of *O*(*n* lg *n*). From the figure, we see that a suitable value for parameter *m* is somewhere around *m* = 10 and *m* = lg *n* if we are seeking to maximise the speedup gain. Note that with the *m* value in this region the percentage of reduction is above 95% for this experiment. In the result section we will conduct our experiments twice, one for *m* = 10 and one for *m* = lg *n* for real datasets. Of course, this makes sense for *n* sizes from a few hundred points and up, which are the most typical cases in real datasets.

Many rules have been suggested to determine the optimal number of bins, commonly for the purpose of computing histograms [10], with some suggesting a number between 5-20 bins. In fact, numerical packages such as Matlab or numpy (Python) accept arguments for specifying the binning algorithm to be used to determine the number of bins; each algorithm uses a different rule. For instance, the ‘sturges’ algorithm gives the number of bins as *m* = lg *n* + 1. Our suggestion for a suitable value of *m* between 10 and lg *m* is consistent with some of these rules (Matlab and R use 10 bins as default).

## Results

The time to compute the convex hull on *n* 2D points is denoted by *t*_*n*_. The time it takes reducing *n* points to *s* points by the preprocessing proposed here is denoted by *t*_*r*_. The time to compute the convex hull on *s* 2D points is denoted by *t*_*s*_. The speedup achieved by preprocessing 2D points by the method proposed here is then calculated as:

$$\begin{array}{c} speedup=\frac{t_{n}}{t_{r}+t_{s}} \end{array}$$

This section shows the amount of speedup for computing the convex hull through the preprocessing on various datasets. As previously stated we use the same four datasets of our previous work [6] where it was shown that the widely accepted preprocessing method in [5] discards roughly half the points.

### Mammal dataset

**Fig 5:**
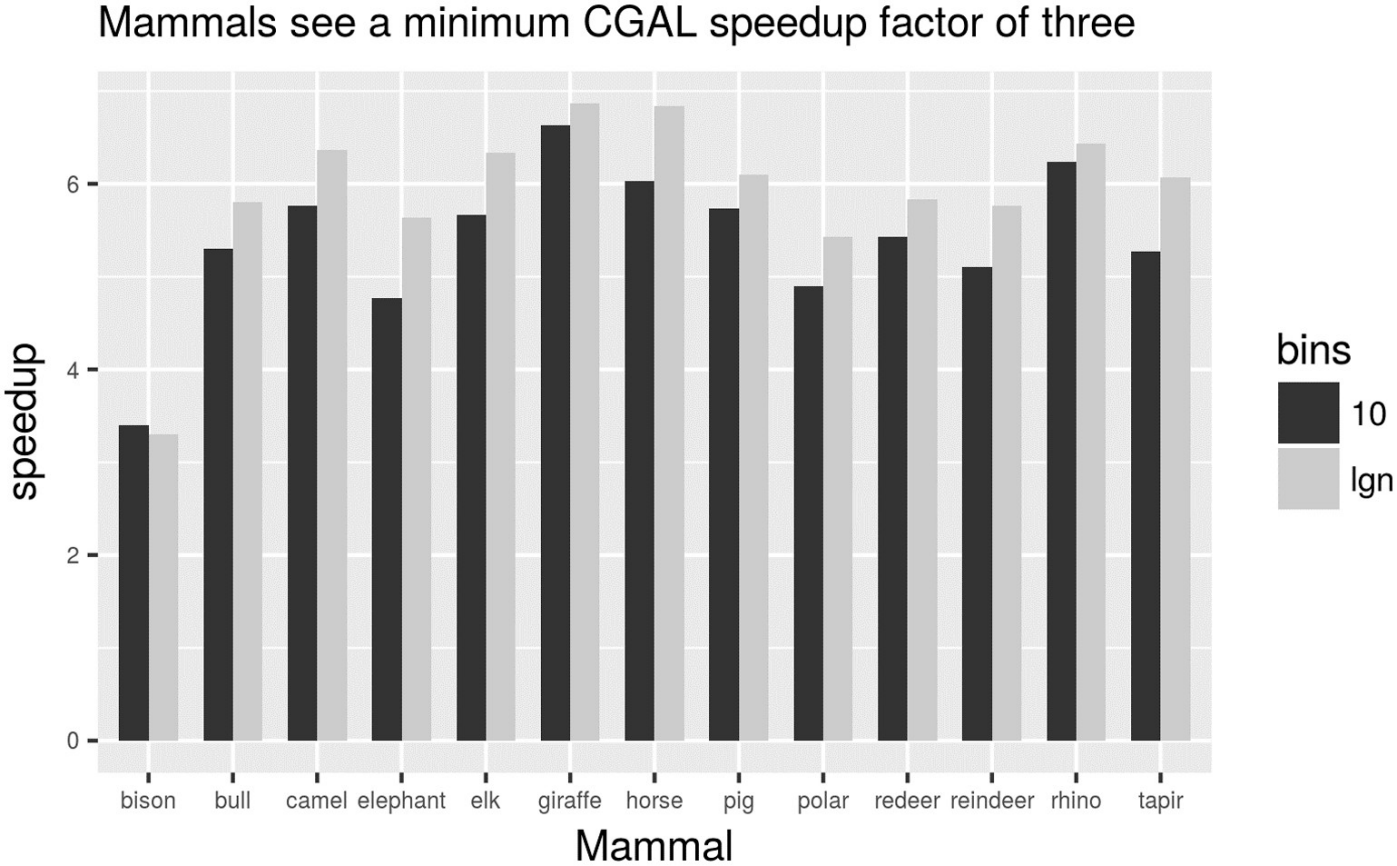
Speedup gained by the reduction method using CGAL. Bar ‘10’ is for parameter *m* = 10 while bar ‘lgn’ is for *m* = lg *n*; this *m* value is the number of bins used in Step 2 of the proposed preprocessing method.

This dataset is from a 3D scan of points of 13 skeletons of mammals [11]; each mammal with a data size ranging from around half million points to 5 million points. Mammals’ scans include a bull (1.4M points), a horse (1.7M points), a reindeer (0.8M points), a giraffe (2.2M points), an elephant (4.9M points) etc. They generate convex hulls of around 20-60 points each. The speedup has been calculated as the average of the convex hull running times for the three 2D projections for each mammal data points (that is keeping (*x*, *y*), (*x*, *z*) and (*y*, *z*) coordinates of the 3D points). The convex hull was computed using the akl_toussaint algorithm as available from the CGAL package. Fig 5 shows the speedup in the convex hull computation for the cases of 10 bins and for *m* = lg *n* number of bins.

**Fig 6:**
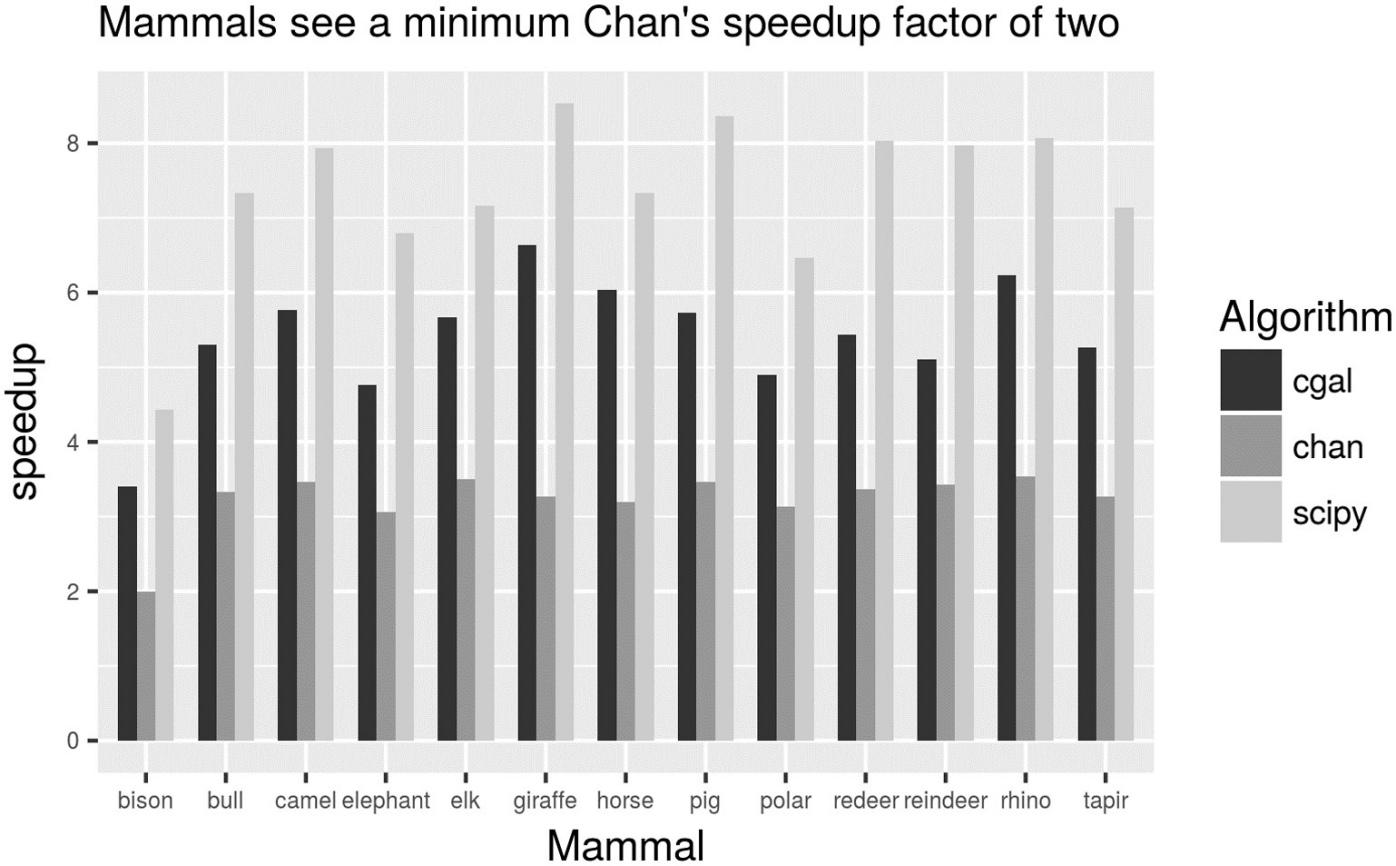
Speedup gained by the reduction method for three convex hull algorithms. Bar ‘cgal’ is for from CGAL package as before, bar ‘scipy’ is from convex hull found in SciPy package [8] and bar ‘chan’ is from an ad-hoc implementation of the Chan algorithm. Speedup is reported for *m* = 10 bins.

As the difference in speedup between *m* = 10 and *m* = lg *n* in Fig 5 is of around 10%, Fig 6 only shows the speedup for *m* = 10 across three different convex hull algorithms. Note that the algorithm due to Chan is not commonly found in computational geometry packages and so we have used an implementation of the algorithm as available from [12]. Even though this ad-hoc implementation of Chan is probably not optimised for production, the proposed preconditioning method offers an speedup of at least a factor of two for this dataset.

### Homerange dataset

**Fig 7:**
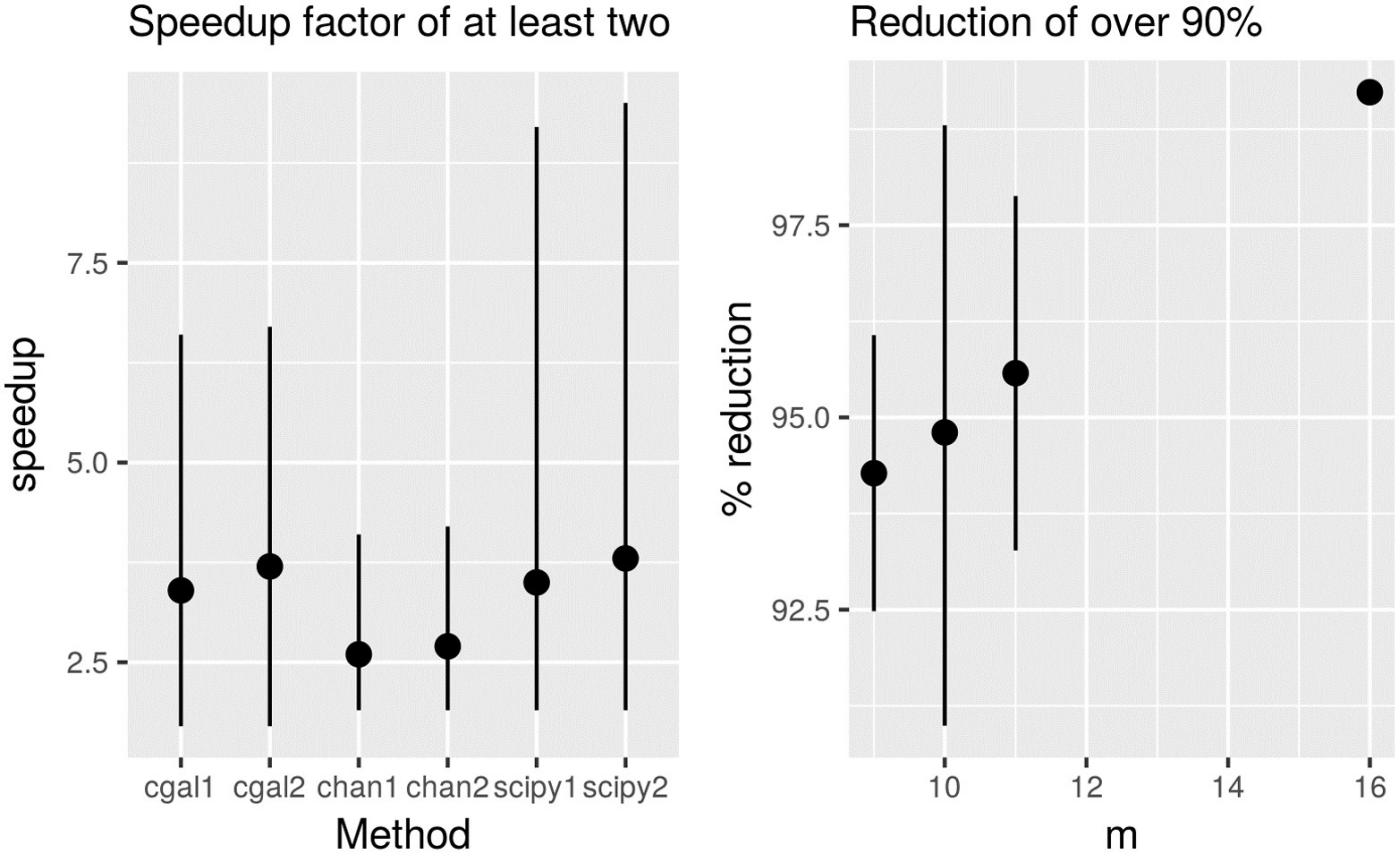
Speedup factor and percentage of reduction of time for three convex hull algorithms for a homerange dataset. Label ‘Method-1’ (for instance cgal1) corresponds to *m* = 10 while ‘Method-2’ (for instance cgal2) corresponds to *m* = lg *n*. ‘cgal’ is from CGAL package, ‘scipy’ is from SciPy package [8] and ‘chan’ is from an ad-hoc implementation of the Chan algorithm. The thin bars represent the distribution of speedup provided by the preprocessing for each algorithm across all the wildlife species; from minimum to maximum (median as a dot). On the right, the bars represent the distribution of the percentage of reduction across all species, except at *m* = 16 that is the median (of the reduction) of all species combined.

We have used data from the Biodeversity & Wildlife section of the Kenya GIS Data (Geographic Information Systems) [13]. In particular these are spatial distribution of wildlife species (including buffalos, elephants, giraffes, etc.) as observed from low-altitude flights in Kenya across a number of years in the 70’s and 90’s; this spatial distribution of wildlife is their homerange. The number of observations (2D points) of individual species is not very large, typically under 1000 observations; these are of wildebeest, zebras, buffalos, etc. The convex hulls are of around 10-50 points. Note this dataset is sparse and not dense as the mammal dataset, and it is small. Each point has been transformed from CRS (Coordinate Reference System) 32637 (used for topographical mapping for a strip area of the world containing Kenya) to CRS 4326 (basically lattiute, longitude coordinates for GPS). This dataset also includes all species combined for different years (with names ke_wildlife_1970 and ke_wildlife_1990 from the web site). The left part of Fig 7 shows the speedup gain when using the proposed preprocessing using three convex hull algorithms. Again, a speedup factor of at least two is observed in spite of the small size of *n*. On the right of Fig 7, the percentage of reduction achieved by the proposed method as a function of parameter *m* is shown; this is consistently above 90%.

### Synthetic dataset

**Fig 8:**
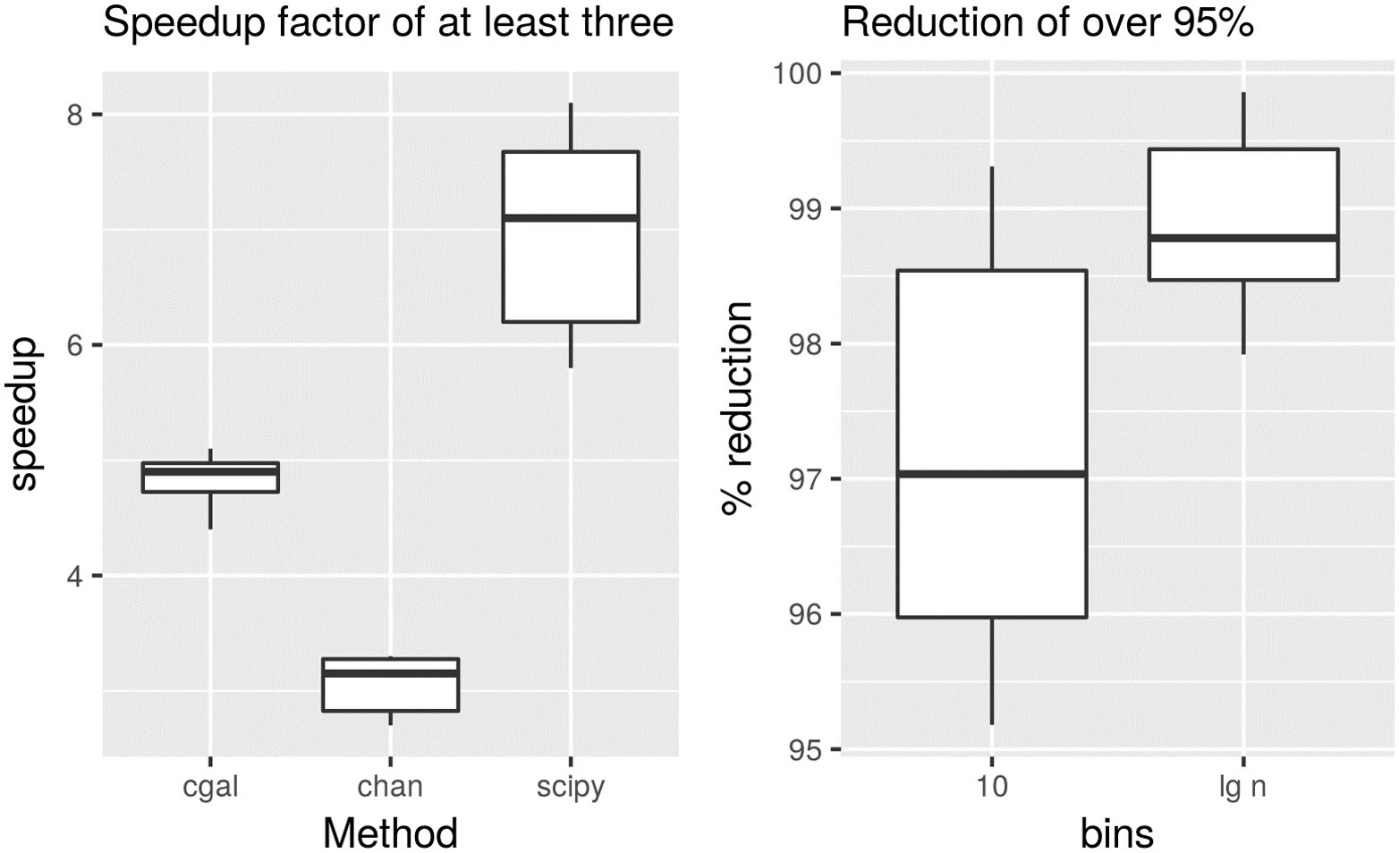
Speedup and percentage of reduction for three convex hull algorithms for a synthetic dataset. On the left, speedup was computed with parameter *m* = 10 for the three algorithms, ‘cgal’, ‘scipy’ and ‘chan’. On the right the percentage of reduction is shown for the case of a constant parameter *m* = 10 and for a variable parameter *m* = lg *n*.

The number of points *n* was varied from 1 million to 10 million, and all these points were inside a convex superellipse. Results were averaged over several runs (for each *n* value) with random parameters for the superellipse. Fig 8 shows the average speedup gained when the preprocessing method was applied before each of the convex hull algorithms. The shape of the superellipses may result in convex hull polygons of over 500 sides in this dataset. Again, the smaller computed speedup was for Chan’s algorithm and it was above a factor of two. On the right of Fig 8, the percentage of reduction achieved by the the preprocessing is shown for this synthetic dataset; it is above 95%.

### Image dataset

**Fig 9:**
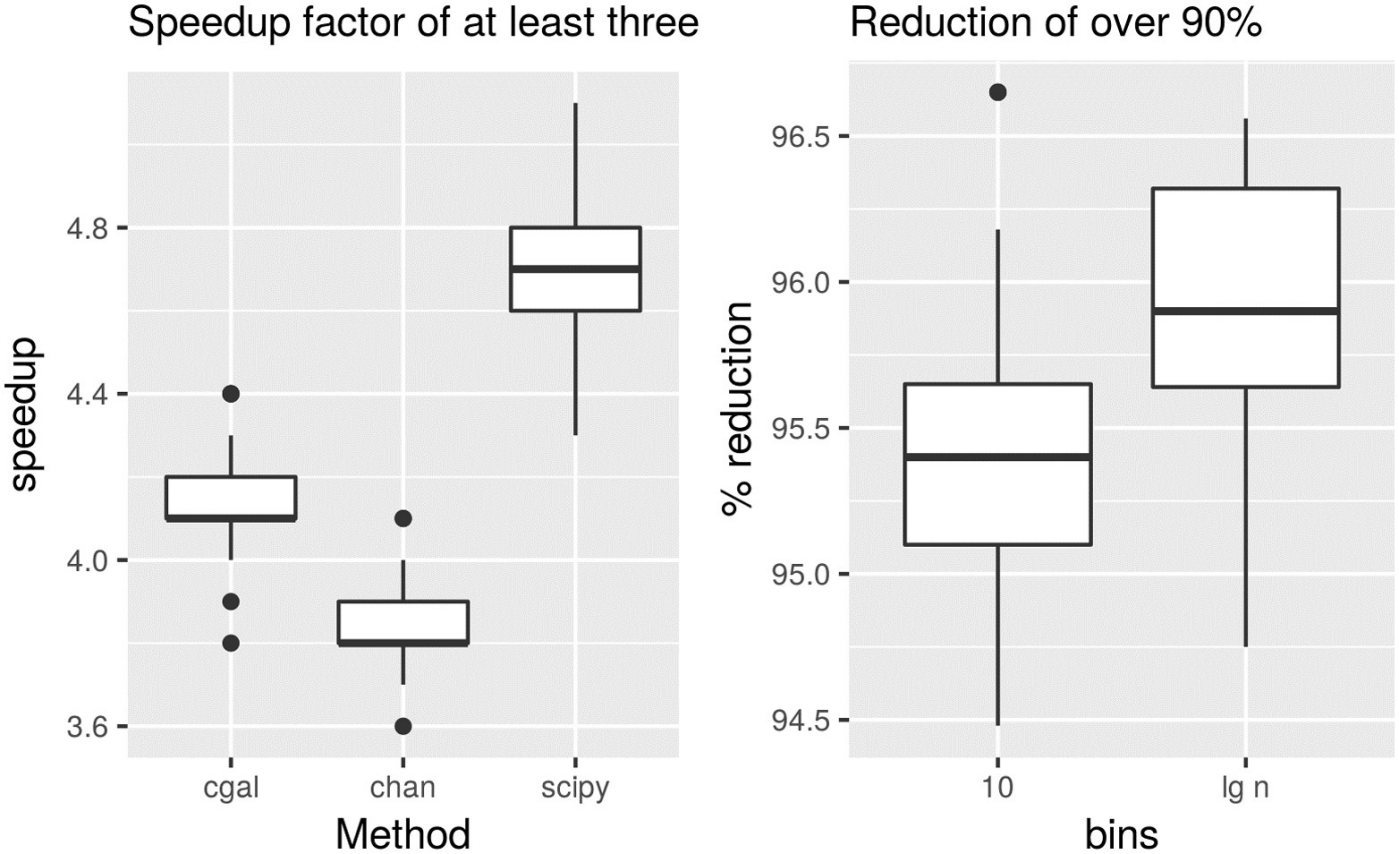
Speedup factor and percentage of reduction of points for three convex hull algorithms for an image dataset. On the left, speedup was computed for a constant parameter value of *m* = 10 for the three algorithms, ‘cgal’, ‘scipy’ and ‘chan’. On the right the percentage of reduction of points is shown for both a constant parameter value of *m* = 10 and for a variable parameter value of *m* = lg *n*. Note all points are integers in this dataset.

This dataset is composed of 49 brain MRI midsaggital planes [14]. Each image is not very large with a number of points of around 5 thousand. Their convex hull is of around 30 points each. But importantly, they are integers; this is to show the method is generic regarding the data representation of points. The speedup obtained across these images is shown on the left of Fig 9, while the percentage in the reduction of points is shown on the right. This time the reduction of points is of over 95% with an speedup factor of at least three.

## Discussion

Our numerical results, in four datasets, show empirical evidence that the proposed preprocessing method for convex hulls offers a reduction of points of over 90% while providing speedups of at least a factor of two when computing the convex hull with the fastest known algorithm. The percentage of reduction of points is above 98% at some instances. We find that parameter value *m*, for binning data used in the method, can be kept constant at a value of 10 or calculated from the data size to around the value of lg *n*. We have computed the speedup as $\frac{t_{n}}{t_{r}+t_{s}}$; that is execution time without preprocessing (*t*_*n*_, convex hull time for *n* points) divided by execution with preprocessing (*t*_*r*_ + *t*_*s*_; time to perform the preprocessing, *t*_*r*_, plus convex hull time for the reduced set of *s* points, *t*_*s*_). The preprocessing method achieves a reduction factor of *p*_*r*_ = 1 − *s*/*n*. One way to explain the speedup seen here is to think in terms of the Amdhal’s law used to measure performance gain in computer architectures [15]. As such, speedup in Amdhal’s law is given by $speedup=\frac{1}{1-f_{enh}+f_{enh}/speedup_{enh}}$ with *f*_*enh*_ as the fraction of the computation that was enhanced and *speedup*_*enh*_ the speedup achieved for that fraction of the computation enhaced. In our case, *f*_*enh*_ = *p*_*r*_ but it is hard to determine the enhanced speedup since the preprocessing method and a convex hull algorithm perform different computational tasks. Nevertheless, for a convex algorithm such as ch_akl_toussaint with a complexity of *O*(*n* lg *n*), theoretically we expect an enhanced speedup of *k* = *c* lg *n* since our preprocessing is of time *O*(*n*). That is, we expect an overall speedup of $\frac{1}{\left(1-p_{r}\right)+\frac{p_{r}}{k}}$ (in this case). Numerically, we could calculate *k* from our experiments as $k=\frac{t_{n}\left(n-s\right)}{n\left(t_{r}+t_{s}\right)-st_{n}}$ and take the average over different runs.

**Fig 10:**
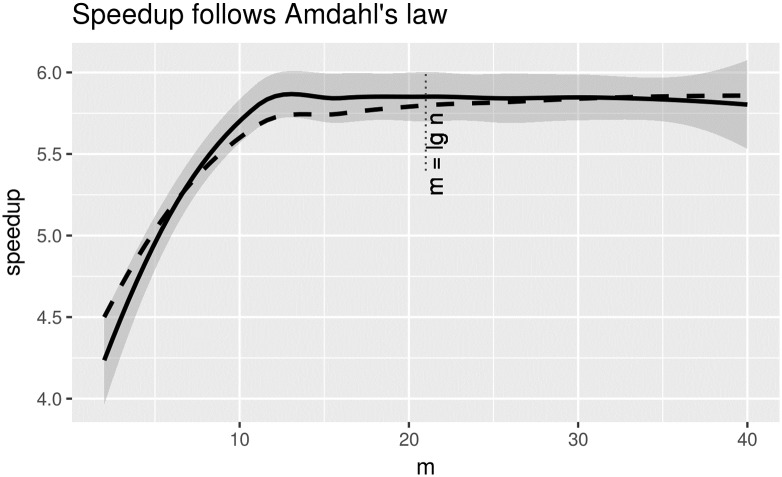
Speedup measured and expected according to Amdhal’s law on the ‘bull’ data from the mammal dataset. The solid line is the measured speedup, while the dashed line is the one predicted from applying Amdhal’s law. For Amdhal’s law an enhanced speedup was calculated as an average over *m* into a constant *k*. CGAL was used to compute the convex hull.

To illustrate this reasoning we used the ‘bull’ data from the mammal dataset which has a calculated value (using the formula above) of *k* = 5.9. Fig 10 shows the actual speedup from the measured *t*_*r*_, *t*_*s*_, *t*_*n*_ running times and the expected speedup according to Amdhal’s law (in terms of *p*_*r*_, *k*); the plot shows this reasoning is helpful to explain the speedup reported here. The preprocessing method, as such, contributes to this speedup by making substantial reductions of points of well over 90%. However, greater speedups can be achieved by larger values of *k*. One obvious way forward, to get a larger *k*, is to exploit the parallelism that is now explicit in some of the steps of the preprocessing method. Steps 4 and 5 process the lower and upper fences independently for example. But it is in Step 6 where much more parallelism is available within each of up to 2(*m* + 1) regions (line segments). In each one of these regions computation can work independently in the process of accepting/rejecting points. Exploring this potential further speedup is left for future work.

Also, Step 6 of the preprocessing method requires quantizing data into a set of bins defined by boundary values whose size is not uniform. This operation is very common in histogramming or for preconditioning data for machine learning. Different quantization methods (binning) could have been explored; this work has opted for the simplest since we are seeking for a fast preprocessing, for other methods, see [16].

This quantization process requires comparing the value to be quantized against the bin boundaries. The common method to establish the bin where a given value belongs to, in a set of *m* bins, is to apply a binary search algorithm per item [1]. That takes *O*(lg *m*) time per item resulting in *O*(*n* lg *m*) for *n* items. If we had chosen binary search for quantizing the data in Step 6, preprocessing would have been of time complexity *O*(*n* lg *m*). In such a case it would not be theoretically faster than Chan’s convex hull algorithm of *O*(*n* lg *h*). Alternatively, binning can be done with fractional cascading [17] having a *O*(lg *m* + *l*) time per item (*l* a constant to be chosen) or with interpolation search with an average time of *O*(lg lg *m*) per item [18]. These alternatives, although much faster than a simple binary search, are still not fast enough for this problem since in general neither *O*(lg lg *m*) < *O*(lg *h*) nor *O*(lg *m* + *l*) < *O*(lg *h*) can be claimed.

## Conclusion

The preprocessing method for a set of 2D points proposed here is general in the sense that it can be applied to points expressed as real or integers. We prove that the method is linear in time, and so it is suitable to be applied in combination with any known convex hull algorithm. Indeed, the results from applying the method in the computation of the convex hull of 2D points, report an speedup of at least a factor of two when using Chan’s algorithm, which is the fastest known convex hull algorithm. Empirical results on small and large datasets, show the method reduces points in an amount well over 90%. As there is little room for improvement based on the reduction of points alone, yet faster speedup factors can be achieved by exploiting parallelism made explicit across the different computational steps as described in the paper; this is left for future work. The preprocessing method given here is simple to implement and can easily be incorporated into computational geometry packages via an on/off flag.
